# Performance comparison of new Veris and Xpert random access HIV-1 RNA quantification assays

**DOI:** 10.1186/s12985-018-1072-x

**Published:** 2018-10-11

**Authors:** Charlotte Pronier, Sarrah Boukthir, Laura Courtellemont, Gisèle Lagathu, Anne Maillard, Vincent Thibault

**Affiliations:** 1 Virology Department, CHU Pontchaillou, Rennes, France; 2 Virology Department, CHU Rennes, Univ Rennes, INSERM, EHESP, IRSET - UMR_S 1085, 2 rue Henri Le Guilloux, 35000 Rennes, France

**Keywords:** Random access testing, Viral load, Genotype, Detection, Quantification, Bias

## Abstract

**Background:**

Recent systems for Human Immunodeficiency Virus 1 (HIV-1) viral load (VL) monitoring allow one-by-one analysis and fast turn-around-time for results. VL measurement on two rapid recently commercialized systems, GeneXpert (Cepheid) and Veris (Beckman Coulter) was compared to classical methods.

**Methods:**

Plasma specimen from HIV-1 (group M) positive patients (*n* = 129) initially quantified with Abbott RealTime HIV-1 and Generic HIV-VL Biocentric assays were retrospectively tested with GeneXpert and Veris.

**Results:**

Valid results on all techniques were obtained for 116/129 specimens composed of 89 Abbott quantifiable VL (38 B, 51 non-B subtypes) [range: 2.09–7.20 log cp/mL] and 27 plasma (9 B, 18 non-B) with Abbott-VL below the limit of quantification (LLQ). All techniques showed good correlation and agreement with a lowest Spearman correlation coefficient of 0.86. Compared to Abbott, the mean bias was 0.35 (95% CI: 0.25–0.45), 0.44 (0.36–0.53) and − 0.04 (− 0.13–0.05) for Biocentric, Beckman and Cepheid, respectively. A difference over 0.5 log cp/mL between VL-quantification of the same sample was observed for 19, 9 and 6 samples with Biocentric, Beckman and Cepheid, respectively. No influence of HIV-1 subtypes on VL was identified. Among 29 samples below LLQ on Abbott, only one was detected and quantified with the Veris assay (38 cp/mL), none with Cepheid.

**Conclusion:**

Both random access systems from Cepheid and Beckman appear well designed for quantifying plasma HIV-1 VL, are easy to handle, fast and fully automated. The slight observed differences suggest to follow the current guidelines recommending the use of the same technique over time for patient viral load monitoring.

## Background

Quantitative real-time PCR (qPCR) is established in many molecular laboratories worldwide and has become an essential tool to monitor various chronic viral infections. HIV-1 Plasma viral load (VL) is a key element for HIV diagnosis in some particular cases such as acute infection or mother-to-child transmission and for the viral follow-up of chronically infected patients to assess efficacy of antiretroviral treatment (ART). VL monitoring, a gold standard practice in resource-rich countries, is indicated at different frequencies before and after initiation of ART or modification whenever a doubt on treatment efficacy is raised, whatever the cause (compliance, resistance emergence). It requires precise and reproducible analysis to ensure optimal monitoring of HIV-infection.

Several commercial assays based on reverse transcriptase-qPCR were developed during the past-decades to quantify plasma HIV-1 RNA. Although current VL technologies share common technical features that make them comparable in terms of analytical performance (lower limit of detection, linear range and HIV subtype detection), they differ in terms of test principles, specimen throughput capacities, costs, infrastructure and human resource requirements. During the last decade, updated versions of these assays led to better sensitivity and genetic exhaustiveness [1]. Other technologies were more recently developed like the GeneXpert (Cepheid) and the DxN Veris systems (Beckman-Coulter) [2]. Compared to other methodologies, they offer a true random access technology allowing to analyze samples on a one-by-one basis throughout the working day.

In this study, we compared Abbott RealTime HIV-1 assay (*m2000sp/m2000rt* Abbott Molecular) which is routinely used in our laboratory along with the Generic HIV-1 viral load assay (Biocentric) for HIV-1 VL monitoring with the Xpert HIV-1 viral load assay on the GeneXpert platform, and the VERIS HIV-1 assay. This extensive comparison was done on a large panel of HIV-1 variants representative of HIV-1 group M genetic diversity.

## Methods

### HIV viral load assays

The RealTime HIV-1 assay (Abbott) combines automated plasma RNA extraction on the *m2000sp* system and real-time PCR amplification of an integrase gene fragment, on the fully automated *m2000rt* PCR system. When using the 0.6 ml plasma protocol the linear detection/quantification range is 40 to 10,000,000 copies per milliliter (cp/mL). The time to results depends on the number of samples per set (24, 48, 72 or 96 samples) with a minimum of 4 h30 from sample to result.

Biocentric Generic HIV-1 viral load assay is a partially manual technique based on RT-PCR amplification within the conserved HIV-1 long terminal repeat (LTR) region, suitable to the majority of PCR real-time opened platforms. In our study, viral RNA extraction was performed manually with QIAmp^®^ Viral RNA Mini Kit (Qiagen). HIV-1 genome was amplified on a StepOnePlus platform (Applied Biosystems). The range of quantification depending on the used protocol is from 50 cp/mL (ultrasensitive technique; with 1 mL of processed plasma) or 300 cp/mL (standard protocol on 200 μL of plasma) up to 5,000,000 cp/mL. The run time depends on the number of samples per set (minimum 3 h).

Cepheid Xpert HIV-1 Viral Load assay is fully automated and combines RNA extraction and purification, reverse transcription, and real-time RT-PCR within the 3′-LTR region, in one integrated cartridge. It runs on the GeneXpert system (Cepheid) on 1 mL input of plasma and has a linear detection range of 40 to 10,000,000 cp/mL. It is designed to work on a one-by-one basis with an acquisition time of 90 min for each sample.

Finally, the VERIS MDx system (Beckman Coulter) is a true random access integrated automated nucleic acid extraction and real-time PCR system that uses a plasma input volume of 0.175 mL or 1.0 mL. Quantification is also based on the 3′-LTR region amplification. The lower and upper limit of quantification are determined to be 35 cp/mL and 10,600,000 cp/mL respectively with the 1-mL assay. The acquisition time is 90 min for 1 patient [3].

### Sample collection

The study included samples from 129 HIV-1 infected patients, collected during routine VL measurements at the University Hospital Pontchaillou of Rennes, France. After collection and centrifugation, K_2_ EDTA plasma samples stored at − 70 °C until testing were initially quantified with both Abbott and Biocentric Generic HIV-1 viral load assays.

After a single freeze/thaw cycle, all 129 samples were tested simultaneously on both GeneXpert and Veris systems. When the volume was insufficient, samples were diluted with HIV negative human plasma (1:2 to 1:5) and the final concentration was calculated according to the performed dilution. Overall and mostly because mishandling of few samples, 116 results were finally available.

The genetic diversity of the panel was determined by Pol region sequencing (protease and reverse transcriptase) according to the ANRS recommendations. The genetic distribution of quantifiable samples was as follows: subtypes A = 9, B = 50, C = 2, F = 19, G = 4, H = 1, Circulating Recombinant Form (CRF)02_AG = 15, CRF37_cpx = 1, non typable (NT) = 17. Subtype F samples were tested to specifically explore subtype F HIV RNA quantification differences suspected on preliminary data.

### Inclusion criteria

A random selection of specimens from adults over 18 years of age, covering the entire range of quantification and with sufficient volume to perform the four described techniques, were selected from our routine work.

### Statistical analysis

All results were transformed to log_10_ cp/mL for further statistical analysis. The number of samples with inter-assay differences exceeding the clinical cutoff value of 0.5 log cp/mL was also recorded. Indeed, fluctuation of HIV-1 RNA VL over 0.5 log cp/mL are considered significant changes that require medical attention such as treatment adjustment [4]. Passing–Bablok regression and Bland–Altman analyses were performed [5]. To determine the linear relationship between two assays, the Spearman correlation coefficient (*r*) was calculated. Means of all differences and standard deviations (SDs) were calculated. The 95% limits of agreement between assays were determined as the mean ± 1.96 SD.

## Results

Results from all four assays were available for 116 samples: 89 plasmas with HIV-1 RNA quantified with Abbott and Biocentric assays and 27 samples with a viral load not quantified with the Abbott assay. Eighty-nine samples were quantified with the Abbott and the Biocentric assays with a quantification range of 2.09–7.20 log cp/mL and of 2.43–8.18 log cp/mL respectively.

The 27 samples not quantified with Abbott included samples below Abbott assay quantification range (< 40 cp/mL; *n* = 27), giving a result of either target detected (*n* = 13) or not detected (*n* = 14) (Table 1).

**Table 1:** Comparison of Beckman and Cepheid assays versus Abbott for the 27 not quantifiable samples

		HIV-1 RealTime Abbott	
		Detected (*n* = 13)	Not detected (*n* = 14)
Beckman	quantifiable	0	1 (38 cp/mL)
	detected	9	6
	not detected	4	7
Cepheid	detected	6	9
	not detected	7	5

**Fig. 1:**
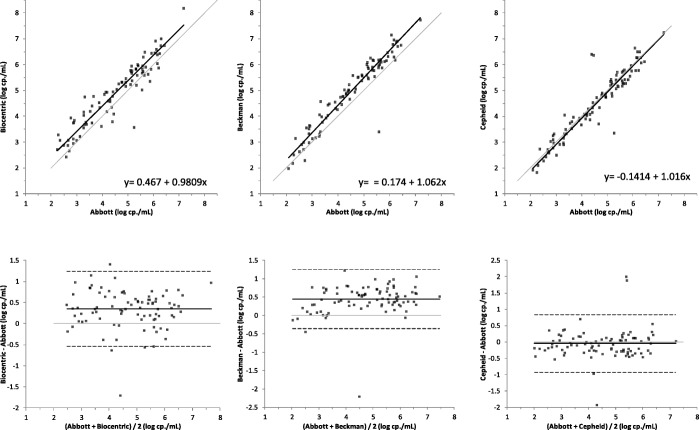
Correlation and Bland-Altman graphs comparing the quantification of HIV-RNA by different methods with HIV-1 RealTime (Abbott). Panels from left to right represent the comparison with, Biocentric, Beckman and Cepheid

An overall good correlation of HIV-RNA quantification by these four assays was observed, with respective nonparametric Spearman rank test (rs) of 0.930, 0.948 and 0.926 for Biocentric, Beckman and Cepheid when compared to Abbott. The lowest value was observed between Beckman and Biocentric rs = 0.876 (Fig. 1). Interestingly, the difference of quantification observed between Abbott and the other systems was constant throughout the quantification range except for Beckman (Bland-Altman, Fig. 1). In this last comparison, the difference tended to be reduced for the lower viral loads.

The assays were further evaluated and compared to our routine results through the Bland-Altman method (Fig. 1). Compared to Abbott, the mean bias for Biocentric was 0.35 (95% CI: 0.25–0.45), it was 0.44 (95% CI: 0.36–0.53) for Beckman and − 0.04 (95% CI: -0.13-0.05) for Cepheid.

**Fig. 2:**
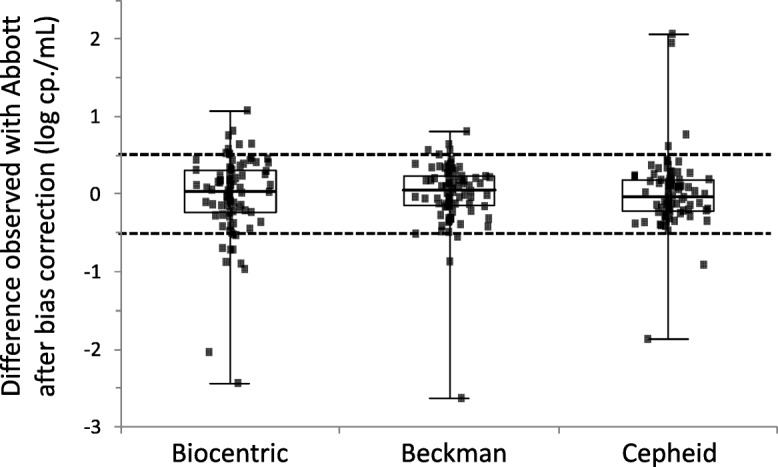
Distribution of the difference of quantification with each technique compared to Abbott values and after adjustment for each technique’s bias. Values above or below 0.5 log cp./mL are considered discordant

After adjustment of the viral loads according to the observed biases for each method, a clinically significant inter-assay difference above 0.5 log cp/mL was observed between assays for few samples. There were 19 discordant samples between Abbott and Biocentric; 9 samples between Abbott and Beckman and 6 samples between Abbott and Cepheid (Fig. 2).

No influence of HIV-1 subtypes on viral load quantification was identified in this set of samples.

Among the 27 samples below the limit of quantification with the Abbott assay, none was quantifiable with Cepheid, but one sample was RNA detected and quantifiable with Veris (VL = 38 cp/mL) (Table 1).

## Discussion

New qPCR platforms offer an innovative approach in the monitoring of viral loads as they allow true random access for molecular biology, in a similar way as what has been used for decades on serological assays. In this study, we demonstrate that the new random access systems offer performances comparable to previous devices requiring to work on set of samples. Our comparison of GeneXpert (Cepheid) and Veris (Beckman Coulter), two recently developed tools offering a one-by-one test approach with quick turn-around time, to a known robust system (m2000sp/m2000rt from Abbott) and to Biocentric, a robust and opened assay, is unique.

Although discrepant quantification between assays had been observed in the past, one can be pleased to observe an excellent correlation between the four assays in this study with Spearman R coefficients ranging from 0.876 to 0.948 [4]. These results confirm the findings of other authors when individually comparing each technique. For instance, Jordan et al. on a large study reported a strong correlation between Cepheid and Abbott (*r* = 0.985), a value close to the one found in this study but also by others [6, 7]. Most importantly, the mean difference in VL quantification was only 0.04 log cp/mL between both techniques. As pointed out by Jordan et al. in their study, 97.7% of the samples fell within the +/− 0.5 log difference range.

In contrast with the results obtained with Cepheid, a rather important bias of − 0.44 log cp/mL was observed between Abbott and Beckman despite an overall excellent correlation between both techniques (*r* = 0.948). After correction for this bias, one should stress that the quantification difference distribution between both techniques was the smallest (SD = 0.411) when compared to Cepheid (SD of the differences = 0.452) or Biocentric (SD = 0.520) (Fig. 2). This bias seems to indicate an imperfect calibration of the technique as it has been observed by two other studies using different comparative techniques [2, 3]. As pointed out in the study by Braun et al., the bias is not constant throughout the quantification range and increases with the viral load [2]. We have also noticed this tendency on the Bland-Altman analysis with the following relationship: Veris Bias = 0.079 x mean VL + 0.0696 (Fig. 1).

To assess any difference of quantification according to HIV-1 subtypes, our selection comprised several of those most often locally encountered, and no remarkable anomaly of quantification whatever the considered subtype was noticed.

The Biocentric assay was initially developed to provide a cost effective and robust assay to resource limited countries [8]. Our study reveals that this assay possesses performances comparable to those provided by the most recent systems. The limited bias with Abbott (− 0.35 log cp/mL) was constant throughout the quantification range but the difference heterogeneity was slightly higher than for the other techniques (SD of the differences = 0.520). This observation could possibly be explained by the manual extraction protocol linked to this technique introducing some handling variations. Last version of manufacturer’s instructions recommend automated isolation of HIV-RNA. Nevertheless, as demonstrated in many clinical studies, this assay is perfectly suitable for HIV-1 monitoring, particularly in resource limited countries [9].

This study was only based on samples from adults but a recent study indicates that they are also suitable for infant samples [10]. The Veris assay could be difficult to implement for children under a year because of the amount of sample used by this assay; in those case the sample volume can be reduced to 0.175 mL or could be manually diluted, the consequence being a slightly reduced sensitivity. Beside random access and according to our more than 18 months experience, the strength of the Veris system is certainly usage simplicity, rapid time-to-result, little hands-on time and reproducibility. Technical evolution for viral load monitoring will inexorably lead to the emergence of such systems in the near future as is observed with the recently developed Aptima HIV-1 Quant Dx assay from Hologic [11]. The possibility to work on a one-by-one basis for molecular biology markers is slowly changing our lab organizations.

## Conclusions

The Veris and Xpert assays are closed and totally automated systems for the quantification of HIV-1 RNA. These two new assays are easy to use and allow quick measurement of infected patient HIV-1 viral load of patients without the need of deep expertise in molecular biology. These systems could improve the follow-up of HIV infected patients. Indeed, shorter time-to-result may promote a better treatment adjustment and could reduce loss to follow-up particularly in resource-limited countries. They also avoid human errors, improve sample traceability and increase workflow efficiency [12, 13]. Yet, observation of few discordant results between the different assays supports the advice to keep monitoring the patient with a unique technique as much as possible.

## References

[1] Damond F, Avettand-Fenoel V, Collin G, Roquebert B, Plantier JC, Ganon A (2010). Evaluation of an upgraded version of the Roche Cobas AmpliPrep/Cobas TaqMan HIV-1 test for HIV-1 load quantification. J Clin Microbiol.

[2] Braun P, Delgado R, Drago M, Fanti D, Fleury H, Hofmann J (2017). A European multicientre study on the comparison of HIV-1 viral loads between VERIS HIV-1 assay and Roche COBAS® TAQMAN® HIV-1 test, Abbott RealTime HIV-1 assay, and Siemens VERSANT HIV-1 assay. J Clin Virol.

[3] Braun P, Delgado R, Drago M, Fanti D, Fleury H, Hofmann J, Caliendo AM (2017). European multicenter study on analytical performance of Veris HIV-1 assay.

[4] Senechal B, James VLA (2012). Ten years of external quality assessment of human immunodeficiency virus type 1 RNA quantification. J Clin Microbiol.

[5] Bland JM, Altman DG (1986). Statistical methods for assessing agreement between two methods of clinical measurement. Lancet Lond Engl.

[6] Jordan JA, Plantier JC, Templeton K, Wu AHB (2016). Multi-site clinical evaluation of the Xpert(®) HIV-1 viral load assay. J Clin Virol Off Publ Pan Am Soc Clin Virol..

[7] Gueudin M, Baron A, Alessandri-Gradt E, Lemée V, Mourez T, Etienne M, Plantier JC. Performance Evaluation of the New HIV-1 Quantification Assay, Xpert HIV-1 Viral Load, on a Wide Panel of HIV-1 Variants. J Acquir Immune Defic Syndr. 2016;15;72(5):521–6. 10.1097/QAI.0000000000001003 27007866

[8] Steegen K, Luchters S, De Cabooter N, Reynaerts J, Mandaliya K, Plum J (2007). Evaluation of two commercially available alternatives for HIV-1 viral load testing in resource-limited settings. J Virol Methods.

[9] Rouet F, Ekouevi DK, Chaix M-L, Burgard M, Inwoley A, Tony TD (2005). Transfer and evaluation of an automated, low-cost real-time reverse transcription-PCR test for diagnosis and monitoring of human immunodeficiency virus type 1 infection in a west African resource-limited setting. J Clin Microbiol.

[10] Ceffa S, Luhanga R, Andreotti M, Brambilla D, Erba F, Jere H (2016). Comparison of the Cepheid GeneXpert and Abbott M2000 HIV-1 real time molecular assays for monitoring HIV-1 viral load and detecting HIV-1 infection. J Virol Methods.

[11] Hopkins M, Hau S, Tiernan C, Papadimitropoulos A, Chawla A, Beloukas A (2015). Comparative performance of the new Aptima HIV-1 quant dx assay with three commercial PCR-based HIV-1 RNA quantitation assays. J Clin Virol.

[12] Espy MJ, Uhl JR, Sloan LM, Buckwalter SP, Jones MF, Vetter EA (2006). Real-time PCR in clinical microbiology: applications for routine laboratory testing. Clin Microbiol Rev.

[13] Cobb BR, Vaks JE, Do T, Vilchez RA (2011). Evolution in the sensitivity of quantitative HIV-1 viral load tests. J Clin Virol Off Publ Pan Am Soc Clin Virol.

